# Electrocardiographic abnormalities and dyslipidaemic syndrome in children with sickle cell anaemia

**DOI:** 10.5830/CVJA-2015-059

**Published:** 2016

**Authors:** Samuel Ademola Adegoke, John Akintunde Oladotun Okeniyi, Adeseye Abiodun Akintunde

**Affiliations:** Paediatric Haematology Unit, Department of Paediatrics and Child Health, Obafemi Awolowo University, Ile-Ife, Nigeria; Paediatric Cardiology Unit, Department of Paediatrics and Child Health, Obafemi Awolowo University, Ile-Ife, Nigeria; Cardiology Division, Department of Medicine, Ladoke Akintola University of Technology, Ogbomoso, Nigeria

**Keywords:** children, dyslipidaemia, electrocardiogram, sickle cell anaemia

## Abstract

**Background:**

Lipid and electrocardiographic (ECG) abnormalities have been reported in adults with sickle cell anaemia (SCA) and may reflect underlying structural and/ or functional damage. However, the relationship between ECG and lipid abnormalities among children with sickle cell disease is not fully understood.

**Objectives:**

To compare the steady-state lipid and ECG abnormalities in children with SCA to the controls and examine the hypothesis that lipid abnormalities are closely related to electrocardiographic abnormalities, and therefore are a reflection of cardiac damage among these children.

**Methods:**

Clinical, laboratory and ECG profiles of 62 children with SCA and 40 age- and gender-matched haemoglobin AA controls were compared. The influence of clinical characteristics, lipids profiles, markers of haemolysis, and renal and hepatic dysfunction on ECG pattern in children with SCA was then determined.

**Results:**

The patients had lower average diastolic and mean arterial blood pressure, total cholesterol and low-density lipoprotein cholesterol (LDL-C) levels than the controls, (*p* = 0.001, 0.002, 0.000 and 0.000, respectively). The mean triglyceride level was significantly higher (*p* < 0.001), while high-density lipoprotein cholesterol (HDL-C) levels were comparable (*p* = 0.858). The cases were about six times more likely to have left ventricular hypertrophy than the controls (OR = 6.4, 95% CI = 2.7–15.6, *p* = 0.000). Haematocrit level had a negative correlation with QT_C_ (*r* = –0.3, *p* = 0.016) and QT intervals (*r* = – 0.3, *p* = 0.044). Triglyceride levels had a positive correlation with the PR interval (*r* = 0.3, *p* = 0.012), while serum alanine transferase (ALT) concentrations had an inverse correlation with PR interval (*r* = –0.3, *p* = 0.015). There was no statistical difference in the sociodemographic and clinical characteristics of the SCA children with or without ECG abnormalities. However, the mean triglyceride and serum ALT levels in those with ECG abnormalities were significantly higher than those without (*p* = 0.007 and 0.045, respectively).

**Conclusion:**

Lipid and ECG abnormalities are common in children with SCA. Elevated triglyceride and serum ALT levels are possible biochemical markers of ECG abnormalities in these patients.

## Background

Several specific and non-specific electrocardiographic abnormalities have been reported in adult patients with sickle cell anaemia (SCA).^1^-^3^ Left ventricular hypertrophy (LVH), the most commonly reported ECG abnormality, has a prevalence ranging from 50 to 75% among different study populations.^3^ Also, significant prolongation of QRS duration, PR and QT_C_ intervals, P wave, QRS and QT_C_ dispersions, as well as T-wave inversion in the right precordial leads have been reported among Nigerian adults with SCA.^1^ Apart from the underlying pathologies, these high-voltage recordings have been attributed to reduced skin fat and thin chest wall in patients with sickle cell disease.^4^

Dyslipidaemic syndrome, characterised by hypocholesterolaemia, hypertriglyceridaemia and reduced plasma high-density lipoprotein cholesterol (HDL-C) levels, is a known metabolic disorder in adults and children with SCA.^5^-^7^ Low total cholesterol in SCA has been linked to chronic haemolysis and/or increased erythropoesis, with a subsequent increase in cholesterol utilisation.^6^ Plasma lipid levels have also been reported to correlate well with biomarkers of vascular haemolysis such as haematocrit level, haemoglobin concentration and lactate dehydrogenase levels in children with SCA.^8^ However, the influence of dyslipidaemic syndrome on the overall severity and development of electrocardiographic (ECG) abnormalities in children with sickle cell anaemia is not fully understood.

‘Nature’ and ‘nurture’ are known to influence SCA severity and the development of complications.^9^ Some of these factors include the patient’s environment; genetic modifiers, especially β-globin gene haplotype and foetal haemoglobin levels; and several other haematological and biochemical markers, including serum lipids and lipoproteins. These markers, in addition to determining the severity of SCA, also help to predict the possible complications a patient with SCA may develop.^9^ Therefore, a search for potential biomarkers of SCA disease severity would contribute positively to overall SCA management.

In addition to comparing the steady-state lipid profiles of children with SCA with suitable controls and determining the prevalence of ECG abnormalities, this study examined the hypothesis that lipids are potential biochemical markers of ECG abnormalities in sickle cell disease. To achieve this, we related the clinical, haematological and biochemical profiles, including the steady-state lipid profiles, of children with SCA with their ECG pattern.

## Methods

This was a cross-sectional, case–control study of the cohort of children with sickle cell anaemia attending the paediatric haematology clinic of Wesley Guild Hospital, Ilesa Unit, Obafemi Awolowo University Teaching Hospital, Ile-Ife. Cases were consecutive children with SCA (confirmed by haemoglobin electrophoresis) aged two to 15 years in steady state. The age limits were set at 15 and two years, as the older children are managed in our hospital in the adult haematology clinic, and those younger than two would not be old enough to cooperate during electrocardiography.

Steady state was defined as a period without any acute event such as pain, fever, infection or severe anaemia, and no transfusion in the four weeks preceding recruitment.^10^ Controls were age- and gender-matched apparently healthy haemoglobin AA children who attended the children’s welfare clinic of the hospital for pre-school-entry medical tests. Children with SCA on hydroxyurea, or those with known congenital or acquired heart disease, as well as controls with any acute illness in the previous two weeks were not incuded in the study. Also, none of the subjects were on medications known to prolong QT_C_ interval, such as halofantrine and anti-histamine. Parents of all participants agreed to and signed written, informed consents before commencing the study.

Data on sociodemographic characteristics (age, gender, socioeconomic class) and age at diagnosis were obtained by structured questionnaires. Socio-economic class was determined using the occupation of the father and the highest academic qualification of the mother, as described by Olusanya *et al.*^11^ Severity of SCD was assessed using frequency of significant painful episodes, blood transfusions and SCD-related hospitalisation in the previous 12 months, and history of complications.

The children’s weights (kg) were measured using the SECA® electronic scale with an accuracy of 0.1 kg, with subjects standing upright, barefoot and wearing only light clothing. Heights (cm) were measured with a fixed stadiometer, Spirit Height®, with the children standing erect and barefoot. From the measured values of weight and height, the body mass index (BMI) was calculated (kg/m^2^).^12^

Liver and splenic enlargement were assessed clinically and documented as size (cm), palpable from the corresponding costal margins, vertically along the mid-clavicular line, using an inelastic tape measure.^13^ Blood pressures (BP) were taken supine using the Accuson® mercurial sphygmomanometer. The average of two readings was documented in mmHg. The systolic BP corresponded to the first Korotkoff sound while the diastolic BP corresponded to the fifth Korotkoff sound.^14^

The lipid profiles were determined using CardioMetabolic® Profile 1 test kits to obtain total cholesterol (TC), HDL-C and triglyceride (TG) levels. Low-density lipoprotein cholesterol (LDL-C) levels were calculated using the Friedwald equation.^15^ Haematocrit, platelet and total leucocyte counts, serum bilirubin, creatinine levels, total protein, albumin, aspartate transferase (AST), alanine transferase (ALT) and alkaline phosphatase assays were done for the cases using standard methods.

All the participants were evaluated with 12-lead electrocardiography, which was performed using the Biocare® IE-12A model digital electrocardiography machine at a paper speed of 25 mm/s and standardised at 0.1 mV/mm. One of the authors (JAOO) performed and analysed all electrocardiograms. Measurements of the heart rate, cardiac axis, PR interval, QRS duration and QT_C_ interval were done in the standard fashion, as previously described.^16^,^17^ Electrocardiographic reference values for Nigerian children were used as cut-off values for the duration of electrocardiographic deflections and intervals.^17^ Sokolow and Lyon voltage criteria was used to determine LVH on ECG.^18^

## Statistical analysis

The clinical, laboratory and ECG profiles of cases and controls were summarised and presented as proportions and percentages for categorical data and means ± standard deviation (SD), and median and range for continuous data. Categorical variables were compared using the chi-squared or Fisher’s exact tests, while metric data were compared with the independent samples *t*-test, analysis of variance (ANOVA) or Pearson/Spearman correlation test as indicated; *p*-values < 0.05 were taken as statistically significant.

## Results

A total of 102 children, comprising 62 homozygous SS cases and 40 age- and gender-matched haemoglobin AA controls, were recruited for this study. The overall male:female ratio was 1.4:1. Their ages ranged from two to 15 years, with a mean ± SD of 7.76 ± 3.66 years.

The sociodemographic characteristics and anthropometric measurements of the cases and controls were similar (Table 1). However, the cases had lower mean diastolic blood pressure and mean arterial pressure than the controls (*p* < 0.05) (Table 2).

**Table 1:** Baseline characteristics of the cases and controls

*Baseline characteristics*	*Cases (n = 62)*	*Controls (n = 40)*	*p-value*	*95% CI*
Male gender	39 (62.9)	21 (52.5)	0.297	0.7–3.4
Age (years), mean ± SD	7.77 ± 3.87	7.75 ± 3.35	0.974	1.4–1.5
Median age (range)	7.0 (2–16)	8.0 (2–14)		
Age 2–5 years, n (%)	18 (29.0)	13 (32.5)		
Age 6–10 years, n (%)	31 (50.0)	18 (45.0)	0.883	NA
Age > 10 years, n (%)	13 (21.0)	9 (22.5)		
Upper class, n (%)	18 (29.0)	16 (40.0)		
Middle class, n (%)	19 (30.6)	11 (27.5)	0.507	NA
Lower class, n (%)	25 (40.3)	13 (32.5)		
Weight (kg), mean ± SD	22.90 ± 7.76	26.36 ± 10.85	0.084	–7.4–0.5
Height (m), mean ± SD	1.21 ± 0.20	1.26 ± 0.21	0.313	–0.1–0.04
Pulse pressure (mmHg)	35.97 ± 8.68	30.75 ± 7.56	0.191	–1.6–1.3
Total cholesterol (mmol/l)	2.60 ± 0.42	3.11 ± 0.41	0.000	0.3–0.8
HDL-C (mmol/l)	0.96 ± 0.36	0.95 ± 0.35	0.858	0.1–0.6
Triglycerides (mmol/l)	1.59 ± 0.62	0.99 ± 0.45	0.000	1.4–1.9
LDL-C (mmol/l)	1.27 ± 0.60	1.69 ± 0.48	0.000	0.2–0.6
Total cholesterol:HDL-C	3.11 ± 1.44	3.80 ± 1.60	0.029	1.3–1.7

HDL-C = high-density lipoprotein cholesterol; LDL-C = low-density lipoprotein cholesterol; CI = confidence interval; NA = not applicable.

**Table 2:** Comparison of age-dependent mean electrocardiographic indices and blood pressure parameters between cases and controls

	*2–5 years*		*6–10 years*		*11–15 years*	
*ECG and BP parameters*	*Cases mean (SD)*	*Controls mean (SD)*	*Cases mean (SD)*	*Controls mean (SD)*	*Cases mean (SD)*	*Controls mean (SD)*
HR (beats/min)	109.8 (9.0)	95.6 (13.1)^b^	95.0 (9.6)	90.4 (1.0)^b^	85.4 (1.5)	80.1 (5.1)^b^
PR interval (ms)	141.1 (8.1)	135.9 (1.2)^b^	156.4 (19.3)	142.7 (14.8)^b^	161.7 (30.9)	136.9 (15.7)^b^
QRS interval (ms)	77.2 (8.6)	72.1 (0.4)^b^	86.4 (16.9)	75.2 (5.9)^b^	89.5 (3.5)	81.9 (8.7)^b^
QT interval (ms)	333.2 (16.7)	334.4 (28.2)^a^	355.5 (18.9)	345.2 (20.5)^a^	376.5 (31.2)	360.3 (13.5)^a^
QT_C_ interval (ms)	449.6 (13.4)	421.9 (32.5)^b^	445.4 (16.6)	415.4 (20.3)^b^	451.9 (23.4)	415.7 (14.5)^b^
P axis (°)	42.8 (12.3)	42.4 (19.4)^a^	34.2 (18.9)	41.4 (11.7)^a^	39.5 (22.0)	41.6 (13.2)^a^
QRS axis (°)	43.8 (17.3)	58.0 (22.1)^a^	38.8 (21.6)	54.7 (20.0)^b^	47.9 (14.5)	53.3 (24.9)^a^
T axis (°)	41.2 (13.4)	43.1 (16.7)^a^	38.9 (17.8)	47.7 (11.7)^a^	54.8 (50.5)	42.3 (16.5)^a^
RV5 voltage (mV)	3.1 (1.0)	2.1 (0.9)^b^	3.8 (0.8)	2.5 (0.6)^b^	3.7 (0.9)	2.8 (0.4)^b^
SV1 voltage (mV)	1.7 (0.7)	1.9 (0.6)^a^	1.8 (0.6)	1.4 (0.8)^a^	1.7 (0.4)	1.4 (0.3)^a^
RV5 + SV1 voltage (mV)	4.8 (1.3)	4.0 (0.2)^b^	5.6 (0.8)	3.9 (0.9)^b^	5.4 (1.1)	3.9 (1.4)^b^
SBP (mmHg)	76.4 (11.0)	82.3 (12.2)^a^	89.0 (10.0)	83.3 (13.5)^a^	94.6 (9.9)	95.0 (7.1)^a^
DBP (mmHg)	43.6 (9.0)	54.2 (7.3)^b^	46.3 (8.7)	55.8 (8.9)^b^	52.6 (7.9)	60.0 (7.1)^b^
MAP (mmHg)	54.5 (8.9)	63.6 (8.6)^b^	60.5 (0.3)	65.0 (1.9)^b^	66.6 (1.7)	71.7 (6.7)^b^

SBP = systolic blood pressure; DBP = diastolic blood pressure; MAP = mean arterial pressure;

^a^No significant difference between cases and controls (p > 0.05);

^b^Significant difference between cases and controls (p < 0.05).

While the mean total cholesterol and LDL-C levels were significantly lower among the cases than the controls, the mean triglyceride level was significantly higher among the cases (*p* < 0.001). The mean HDL-C value was however comparable between the two groups (*p* = 0.858). Total cholesterol:HDL-C ratio was also lower among the cases (*p* = 0.029) (Table 1).

Table 2 shows the comparison of age-dependent ECG indices between the cases and controls. The mean ECG-generated heart rate (HR), PR interval, QRS duration and corrected QT interval were higher among children with SCA than the controls (*p* < 0.05). The average RV5 voltage and combined RV5 and SV1 voltages were also higher among the cases (*p* < 0.05), however, the mean QRS axis was lower, while the mean QT intervals were comparable between the two groups.

ECG abnormalities: left ventricular hypertrophy (71.0 vs 27.5%), first-degree atrio-ventricular block (19.4 vs 0%) and T-wave abnormalities consistent with lateral ischaemia (12.9 vs 0%) were significantly more prevalent among cases than controls (*p* = 0.000, 0.008 and 0.021, respectively). Also, children with SCA were about six times more likely to have LVH than ageand gender-matched haemoglobin AA children (OR = 6.4, 95% CI = 2.7–15.6). None of the study participants had left atrial enlargement or T-wave inversion.

There was no statistical difference in the frequency of occurrence of tall T-wave abnormalities, sinus rhythm with ventricular premature complex, right atrial enlargement, right or biventricular hypertrophy, ST depression and conduction anormalies, such as right ventricular conduction delay and non-specific intraventricular conduction block between the two groups. On the other hand, abnormal left-axis deviation and ST-segment elevation were non-significantly less prevalent among children with SCA compared to the controls (Table 3).

**Table 3:** Prevalence of ECG abnormalities among children with SCA and age- and gender-matched haemoglobin AA controls

*ECG abnormalities*	*Cases n (%)*	*Controls n (%)*	*p-value*	*Odds ratio (95% CI)*
Sinus arrhythmia	5 (8.1)	4 (10.0)	0.737	0.8 (0.2–3.1)
Sinus rhythm with ventricular premature complex	1 (1.6)	0 (0)	1.000	2.0 (0.1–47.9)
Right atrial enlargement	3 (4.8)	0 (0)	0.417	1.7 (1.4–2.0)
Left ventricular hypertrophy	44 (71.0)	11 (27.5)	0.000	6.4 (2.7–15.6)
Right ventricular hypertrophy	5 (8.1)	1 (2.5)	0.462	3.4 (0.4–30.4)
Biventricular hypertrophy	3 (4.8)	0 (0)	0.158	1.7 (1.4–2.0)
ST-segment elevation	3 (4.8)	3 (7.5)	0.899	0.6 (0.1–3.2)
ST depression	4 (6.5)	0 (0)	0.264	1.7 (1.4–2.0)
Tall T-wave anomaly	1 (1.6)	0 (0)	1.000	1.9 (0.1–47.9)
T-wave abnormality consistent with lateral ischaemia	8 (12.9)	0 (0)	0.021	12.6 (1.7–21.7)
Non-specific T-wave abnormalities	4 (6.5)	1 (2.5)	0.646	2.7 (0.3–24.3)
Right ventricular conduction delay	1 (1.6)	0 (0)	1.000	2.0 (0.1–47.9)
Non-specific intraventricular conduction block	2 (3.2)	0 (0)	0.519	3.4 (0.2–69.1)
Abnormal left-axis deviation	1 (1.6)	2 (5.0)	0.559	0.3 (0.1–3.5)
First-degree atrio-ventricular block	12 (19.4)	0 (0)	0.008	1.8 (1.5–2.2)
Presence of at least one abnormality on ECG findings	49 (79.0)	18 (45.0)	0.000	4.6 (1.9–10.9)

In all, only 13 (21.0%) of the cases against 22 (55.0%) of the controls had no identifiable ECG abnormalities (*p* = 0.000, OR = 4.6, 95% CI = 1.9–10.9). Those with identifiable abnormalities included about half; 30 (48.4%) with multiple abnormal ECG features and 19 (30.6%) with single abnormal ECG reports. There was no significant correlation between some ECG durations (heart rate, PR, QRS, QT_C_ and QT intervals) and frequency of significant pain episodes, SCD-related hospitalisation or transfusion in the 12 months preceeding the study (*p* > 0.05) in each occasion.

Haematocrit level had a negative correlation with QT_C_ interval (*r* = –0.3, *p* = 0.016) and QT intervals (*r* = –0.3, *p* = 0.044). While liver enzyme AST levels had a moderate positive correlation with heart rate (*r* = 0.5, *p* = 0.025), ALT levels had a negative correlation with heart rate (*r* = –0.3, *p* = 0.000) and PR interval (*r* = –0.3, *p* = 0.015). Albumin values were positively correlated with QRS interval (*r* = 0.5, *p* = 0.013), and triglyceride levels positively correlated with PR interval (*r* = 0.3, *p* = 0.012).

Leucocyte and platelet counts, serum total, direct and indirect bilirubin, alkaline phosphatase, total protein, total cholesterol, HDL-C and LDL-C levels had no significant correlation with heart rate, PR, QRS, QT and QT_C_ intervals in children with SCA.

There were no significant statistical differences in the sociodemographic and clinical characteristics of SCA children with or without ECG abnormalities (Table 4). However, the mean values of triglycerides and serum ALT of SCA children with ECG abnormalities were significantly higher than those with normal ECG patterns (*p* = 0.007 and 0.045, respectively) (Table 5).

**Table 4:** Comparison of the sociodemographic and baseline clinical characteristics of SCA children with ECG abnormalities and those without abnormalities

*Characteristics*	*Normal ECG pattern*	*Single ECG abnormality*	*Multiple ECG abnormalities*	p-value*
Number	13	19	30	
Males, n (%)	9 (69.2)	11 (57.9)	19 (63.3)	0.806
Females, n (%)	4 (30.8)	8 (42.1)	11 (36.7)	
Mean age	7.2 ± 4.3	7.8 ± 3.9	8.0 ± 3.8	0.807
Age 2–5 years, n (%)	6 (46.2)	5 (26.3)	7 (23.3)	
Age 6–10 years, n (%)	4 (30.8)	11 (57.9)	16 (53.3)	0.509
Age > 10 years, n (%)	3 (23.1)	3 (15.8)	7 (23.3)	
Upper class, n (%)	2 (15.4)	9 (47.4)	7 (23.3)	
Middle class, n (%)	5 (38.5)	6 (31.6)	8 (26.7)	0.165
Lower class, n (%)	6 (46.2)	4 (21.1)	15 (50.0)	
≥ 3 pain/12 months, n (%)	6 (46.2)	9 (47.4)	14 (46.7)	0.998
ACS, n (%)	1 (7.7)	3 (15.8)	2 (6.7)	0.576
SBP (mmHg)	84.7 ± 8.5	88.7 ± 12.1	86.0 ± 13.7	0.623
DBP (mmHg)	49.6 ± 8.5	54.2 ± 10.6	48.7 ± 9.4	0.141
MAP (mmHg)	61.3 ± 7.7	65.7 ± 10.4	61.1 ± 10.1	0.247
Pulse pressure (mmHg)	35.0 ± 7.6	34.5 ± 8.3	37.3 ± 9.4	0.487
Weight (kg)	22.4 ± 8.8	24.0 ± 7.7	22.4 ± 7.6	0.774
Height (m)	1.18 ± 0.20	1.24 ± 0.22	1.21 ± 0.20	0.774

*p-values when the three groups were compared; SBP = systolic blood pressure; DBP = diastolic blood pressure; MAP = mean arterial pressure; ACS = acute chest syndrome.

**Table 5:** Comparison of the baseline laboratory profiles of SCA children with and without ECG abnormalities

*Characteristics*	*Normal ECG pattern*	*Single ECG abnormality*	*Multiple ECG abnormalities*	p-value*
Number	13	19	30	
Haematocrit (%)	25.5 ± 4.2	24.4 ± 3.0	23.5 ± 3.8	0.263
Leucocyte count (× 10^3^/mm^3^)	9.27 ± 5.22	9.70 ± 3.69	9.78 ± 6.24	0.964
Platelet count (× 10^5^/ mm^3^)	2.24 ± 0.89	2.33 ± 1.09	2.02 ± 0.39	0.710
Total bilirubin (μmol/l)	32.0 ± 15.3	50.5 ± 16.2	60.5 ± 67.1	0.545
Direct bilirubin (μmol/l)	8.6 ± 4.5	6.9 ± 2.2	16.1 ± 25.4	0.500
Indirect bilirubin (μmol/l)	23.4 ± 11.7	43.6 ± 14.7	44.4 ± 42.7	0.421
Creatinine (mmol/l)	62.6 ± 17.7	66.8 ± 21.3	62.7 ± 21.8	0.858
AST (IU/l)	20.3 ± 17.3	23.8 ± 17.2	22.9 ± 14.6	0.938
ALT (IU/l)	5.0 ± 4.8	10.9 ± 7.1	17.3 ± 9.2	0.045
Alkaline phosphatase	209.7 ± 7.0	236.0 ± 123.8	191.7 ± 131.1	0.815
Total protein (g/dl)	66.8 ± 8.0	69.8 ± 10.6	73.4 ± 8.7	0.472
Albumin (g/dl)	31.8 ± 2.9	36.5 ± 3.1	37.3 ± 8.7	0.334
Total cholesterol (mmol/l)	2.62 ± 0.49	2.52 ± 0.30	2.64 ± 0.46	0.639
HDL-C (mmol/l)	0.94 ± 0.40	1.03 ± 0.35	0.93 ± 0.36	0.665
Triglyceride (mmol/l)	0.74 ± 0.28	0.76 ± 0.25	1.24 ± 0.78	0.007
LDL-C (mmol/l)	1.33 ± 0.41	1.24 ± 0.69	1.27 ± 0.63	0.914
Cholesterol:HDL-C ratio	3.40 ± 1.74	2.78 ± 1.44	3.18 ± 1.31	0.461

*p-values by ANOVA to compare means of the three groups; ALT = alanine transferase; AST = aspartate transferase; HDL-C = high-density lipoprotein cholesterol; LDL-C = low-density lipoprotein cholesterol.

## Discussion

This study examined the ECG abnormalities and lipid profiles of children with SCA. These children had higher levels of triglycerides and lower levels of total cholesterol and LDL-C when compared to suitable age- and gender-matched controls. In addition to higher prevalence of LVH, they also had longer PR intervals, QRS duration, heart rate and corrected QT interval, with the majority (79%) having at least one identifiable ECG abnormality.

Although these findings are not entirely novel for adults with SCA, the demonstration of a positive correlation between triglyceride level and PR interval, as well as higher mean triglyceride levels among SCA children with ECG abnormalities, have not been reported in children. Our finding also supports suggestions that (1) SCA children are at increased risk of developing cardiac abnormalities and, (2) specific dyslipidaemic syndrome, especially elevated levels of triglycerides, is a potential biochemical marker of ECG abnormalities in SCA. As described by Kato *et al.*,^19^ progressive haemolysis-induced vasculopathy, one of the two major subphenotypes associated with clinical and laboratory manifestations of SCA, has been linked to endothelial dysfunction and the subsequent development of reticulocytosis, leg ulcers, priapism, stroke, elevated pulmonary arterial pressure and cardiac abnormalities in sickle cell disease.

The prevalence of ECG abnormalities in children with SCA in this study was 79%. This is comparable to some reported rates among adult Nigerians with SCA.^20^,^21^ Also, the finding of LVH as the ECG abnormality seen in 71% of SCA children is similar to many previous local reports.^20^,^21^ It has also beeen documented previously that Nigerian children with SCA have higher rates of arrhythmias than their counterparts without SCA.^22^ Abnormal loading conditions associated with chronic anaemia lead to chamber dilatation and myocardial remodelling, which progress to ventricular dysfunction.^23^ However, other factors such as genetic variations or polymorphisms are also thought to be involved in the dimensional and functional differences seen in LV dysfunction in SCA.^23^

The findings of T-wave abnormality consistent with lateral ischaemia in children with SCA (12.9 vs 0%, *p* = 0.021) have not been reported previously. This study also demonstrated that PR interval was significantly prolonged, and mean QT_C_ interval was significantly longer in patients with SCA than in the controls. These are consistent with findings by Adebayo *et al.*, Bode-Thomas et al. and Oguanobi *et al.*^1^,^2^,^21^ The prolongation of QT_C_ interval, which implies abnormal repolarisation, can be explained by the fact that patients with SCA experience recurrent microscopic infarctions of the myocardium, especially with repeated vaso-occlusion.^24^

Bode-Thomas and co-workers have demonstrated that ECG changes consistent with myocardial ischaemia are common in children with SCA, especially during episodes of severe vasoocclusive crises, acute chest syndrome, and in those with elevated pulmonary arterial pressure.^25^,^26^ This may actually predispose them to increased risk of cardiovascular mortality from cardiac arrhythmias. Areas of micro-infarction are potential arrhthmogenic sites with the possibility of generating malignant arrhythmias such as ventricular and atrial tachyarrhthmias.

In this study, haematocrit levels had a negative correlation with both QT and QT_C_ intervals in children with SCA. Prolonged and shortened QT_C_ on ECG are both known risk factors for sudden cardiac death.^27^,^28^ Although, the exact mechanism of prolonged QT_C_ interval in sudden cardiac deaths in individuals with SCA is largely unknown, it is speculated that chronic anaemia and associated sub-acute cardiac ischaemia may be associated with ventricular repolarisation defects, which ultimately prolong QT_C_ intervals.^28^

Specific dyslipidaemic subphenotype, especially elevated triglyceride levels, in addition to having a positive correlation with PR interval, was significantly higher among SCA children with ECG abnormalities in this study. Studies have shown that elevated triglyceride levels independently predicted the development of coronary artery disease and myocardial infarction.^23^ Also, it was reported recently that an elevated level of triglycerides, which is already known to be linked with endothelial dysfunction, is an independent predictor of pulmonary hypertesion in patients with SCA.^29^ Raised triglyceride levels is an important cardiovascular risk factor for atherogenesis, as other abnormalities of the lipid profile are much more likely and are a readily available complement to atheromatous plaque formation and progression.

Our study was limited by the small sample size, which may have affected proper data interpretation and the overall generalisability of the findings. Also, fasting samples for lipid estimations were not taken. However, non-fasting lipid levels have been significantly correlated with fasting triglyceride levels, and there is new evidence to show that triglyceride levels measured in the fasting or non-fasting state are important in determining the prognosis of cardiovascular diseases.

## Conclusion

This study showed that lipid and electrocardiographic abnormalities were common among the children with SCA attending the paediatric out-patient clinic of WGH, Ilesa, and they were closely related to the cardiovascular risk of these patients.
